# Methods of Computational Modelling in Studies of Transcranial Direct Current Stimulation (tDCS) in Adults to Inform Protocols for Tinnitus Treatment: A Scoping Review

**DOI:** 10.3390/brainsci16010044

**Published:** 2025-12-29

**Authors:** Kaitlin Tudor, Bas Labree, Rebecca S. Dewey, Derek J. Hoare, Marcus Kaiser, Magdalena Sereda

**Affiliations:** 1 NIHR Nottingham Biomedical Research Centre, Nottingham NG1 5DU, UK; kaitlin.tudor@nottingham.ac.uk (K.T.); bas.labree1@nottingham.ac.uk (B.L.); rebecca.dewey@nottingham.ac.uk (R.S.D.); derek.hoare@nottingham.ac.uk (D.J.H.); marcus.kaiser@nottingham.ac.uk (M.K.); 2 Hearing Sciences, Mental Health and Clinical Neurosciences, School of Medicine, University of Nottingham, Nottingham NG7 2RD, UK; 3 Sir Peter Mansfield Imaging Centre, University of Nottingham, Nottingham NG7 2RD, UK; 4 Precision Imaging, Mental Health and Clinical Neurosciences, School of Medicine, University of Nottingham, Nottingham NG7 2UH, UK; 5 Department of Neurosurgery, Ruijin Hospital, Shanghai Jiao Tong University School of Medicine, Shanghai 200025, China

**Keywords:** transcranial direct current stimulation, computational modelling, finite element modelling

## Abstract

**Background**: Transcranial direct current stimulation (tDCS) involves the application of weak electric currents (typically 0.5–2 mA) via scalp electrodes to promote neuroplastic changes that modulate behaviour or cortical activity. Although there have been promising results in eliminating tinnitus or reducing its loudness or severity, there is also a high degree of inter-individual variability. This may be due to anatomical differences and their influence on the resulting electric field. To optimise and personalise tDCS protocols, computational electric field models based on individual clinical imaging may be utilised to give insight into the induced electric field during tDCS and inform more effective protocols for targeted stimulation. To our knowledge, there are currently no standards for current modelling or reviews which detail the optimal parameters for conducting current modelling studies for tDCS. **Objectives**: The aim of this review is to investigate the methodology of current modelling studies for tDCS so that informed, personalised protocols can be designed by modelling the electric field of the brain during tDCS for tinnitus. By considering the impact of individual anatomical differences on the electric field induced by tDCS, targeted protocols could be developed to reduce tinnitus loudness and severity in a systematic and predictable way. **Design**: The protocol for this review is based on the Preferred Reporting Items for Systematic Reviews and Meta-Analyses Extension for Scoping Reviews (PRISMA-ScR) Checklist. Using online databases, records were identified based on a keyword search for records relevant to current modelling for tDCS, including peer-reviewed papers, clinical trials, the grey literature, theses, dissertations, and conference abstracts. Four thousand two hundred and fifty-three records were retrieved from thirteen online databases and include 4186 records from the initial search completed between May and July 2024, and 67 records from an updated search completed in August 2025. A further 596 records were retrieved from Google Scholar (501 from the initial search and 95 from the updated search). One hundred and fourteen records met our criteria for inclusion. Each record was charted by two separate reviewers, with attention to the modelling pipeline and predicted values in peak and range of electric field magnitude. **Results**: There was a consensus that, despite model parameters and pipelines, there was inter-individual variability in the predicted electric fields. The reviewed records highlighted the impact of individual differences, including age, sex, and anatomical variation, on the predicted electric field during tDCS. Increased age was often associated with age-related brain atrophy and high relative cerebrospinal fluid volume, which was a significant influence on the resulting E-field intensity and distribution. **Conclusions**: When creating personalised tDCS protocols for tinnitus, the model parameters and sources of variability (i.e., morphology, age, and sex) should be carefully considered to achieve the desired stimulation outcomes, particularly in regard to applied current intensity.

## 1. Introduction

Tinnitus is experienced by about 15% of the population [1] and is characterised by the perception of sound in the absence of an external auditory stimulus [2]. This perceived sound can be a significant source of distress and can impact quality of life through its effects on cognitive function, sleep quality, and social participation. Although the exact cause of tinnitus is not completely understood, there are theories to suggest that it develops as a result of maladaptive plastic changes in brain regions of the central auditory system, which process auditory information as a response to auditory deprivation [3]. This central gain theory suggests that the affected regions of the brain generate tinnitus percepts when there is anticipated sound in the absence of an auditory stimulus as a protective mechanism to maintain homeostasis [4]. While there are management strategies such as cognitive behavioural therapy, sound therapy and education [5] available to cope with tinnitus, there is currently no treatment option which would remove the source of tinnitus percepts.

**Figure 1:**
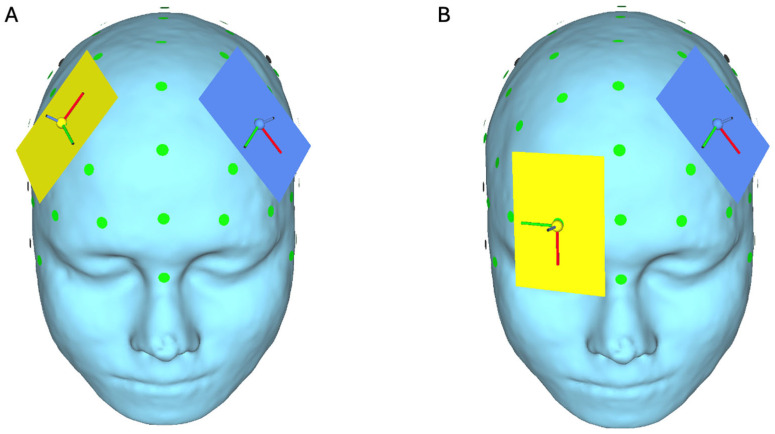
Electrode positions relevant to targeting areas of the brain in tinnitus, demonstrated on the SimNIBS standard head model ‘Ernie’ [15], with (**A**) anode over F3 (blue) and cathode over F4 (yellow) and (**B**) with anode (blue) over F3 and cathode (yellow) over Fp2 in a 10–20 EEG system.

Transcranial direct current stimulation (tDCS) is a promising neuromodulation technique with potential for targeting neural circuits involved in the generation and maintenance of tinnitus. It is currently used for conditions such as depression and stroke recovery [6], but it is not widely available for applications to tinnitus. The technique dates back to bioelectric studies in 43–48 AC when Scribonius Largus used electric torpedo fish for headache [7] and later Galvani’s electrophysiological experiments on nerve conduction and muscle contraction in frogs [8]. The technique has since made a resurgence and an increase in popularity following more recent physiological studies [9] which suggested that tDCS could induce excitability changes that extend beyond the period of stimulation. Weak electric currents are delivered via scalp electrodes to modulate cortical excitability by hyperpolarisation or depolarisation of the neuron [10]. As tinnitus involves maladaptive plastic changes in the central auditory system, tDCS can be used to target these auditory processing areas or the regions involved in maintaining the distress and emotional response to the phantom sounds. Although its use in clinical settings is not widespread, the two most likely effective setups for tinnitus are ones that place the anode over F3 and cathode over F4 (as demonstrated in Figure 1A) and, equally effective, the anode over F3 and cathode placed over Fp2 (as demonstrated in Figure 1B) [11]. The targets of these montages are the dorsolateral prefrontal cortex (DLPFC) and the auditory cortices, the aim of targeting these regions being to disrupt the connectivity between emotional and auditory centres [12].

There is observed efficacy for the use of tDCS for tinnitus [11,12], but its clinical efficacy is limited by variability in current intensity of approximately 50–150% (in the general population) between individuals, regardless of stimulation montage [13]. A key contributor to this variability in outcomes may be the prevalent use of a “fixed dose” approach, wherein all participants receive the same stimulation intensity despite significant differences in the morphology of individuals and the resulting electric field generated by stimulation. Each tissue of the head has different conductive properties, and the volume of each tissue varies between individuals. Some tissues are highly conductive, such as cerebrospinal fluid (CSF), and act as a shunt [14], whereas others are more resistant. This is important to consider as high CSF volume can be correlated with increased age, which could be a potential predictor of stimulation outcomes.

A more informed approach to protocol design could resolve or reduce the impact of inter-individual differences on tDCS outcomes for people with tinnitus, which could make it a clinically feasible option for tinnitus treatment. For tDCS to be a clinically feasible treatment option for tinnitus, its delivery may need to be individualised to improve consistency in clinical outcomes. To achieve this, it may be necessary to plan protocols guided by computational models. These models can be derived from magnetic resonance images (MRI), which provide individual anatomical data to accurately and realistically predict how current would pass through the head as a volume conductor based on the input parameters, the conductivity value of each tissue, and the tissue composition of the individual modelled. Computational modelling enables participant-specific simulation of tDCS-induced electric fields. By segmenting tissue compartments from high-resolution structural MRI data, a mesh can be generated for constructing individualised finite element models of the head. This modelling framework facilitates the personalisation of stimulation protocols, including dose optimisation, to achieve sufficient electric field magnitudes necessary for neuroplastic change in brain regions implicated in tinnitus. Ultimately, such an approach represents a critical step toward more precise, consistent, and effective neuromodulatory interventions. 

Models can be highly customisable due to the number of parameters that can be varied during modelling. However, this array of options also presents the challenge of creating effective modelling protocols. There are decisions to be made regarding the applied stimulation intensity, the electrode montage, electrode orientation, electrode dimensions, the consideration of white matter anisotropy, segmentation techniques, and applied conductivity values. The optimal number and duration of sessions have been investigated previously to conclude that about seven sessions for a duration of 20 min each seems most effective [11]. The applied current intensity for tDCS is often in the range of 1–2 mA [11] but does not seem to be explored in the personalisation of protocols. However, there is some evidence to suggest that doses of cathodal tDCS exceeding 2 mA result in the opposite of the intended effect [16] and that there may be a range in which stimulation is most effective. The non-linear relationship between stimulation effects and dose presents challenges in setting current intensity. However, there are also studies into dose control [13] which may give indications of how to calculate the most effective current intensity for an individual, which will be covered in this review.

Another challenge in optimising tDCS protocols is that there is no defined target intensity for achieving the optimal stimulation at the cortex. It is not known how much current is necessary to drive plastic changes and produce the desired effects of stimulation, which makes it difficult to set a current dose.

### 1.1. Aims

The aim of this review is to provide an overview of the methods reported in computational modelling studies of tDCS for the purpose of developing effective, individualised tDCS protocols for the treatment of tinnitus.

### 1.2. Rationale

To our knowledge, there are currently no reviews that detail the optimal parameters for conducting current modelling studies for tDCS. There are also no reviews that investigate the current modelling of tDCS for the treatment or management of tinnitus. This scoping review should allow for the collation of knowledge of current modelling methods, including the steps and possible approaches reported by the authors, so that informed protocols can be designed for modelling the electric field of the brain during tDCS. In the future, we will be using the results of this review to create personalised protocols guided by current flow models, which can be applied to individuals with tinnitus as a potential treatment option. In particular, we aim to use models to guide and personalise dose control.

This review may also reveal any gaps in the literature where protocols can be optimised or where there is room for exploration in parameters. The review may give indications of how much current is necessary to achieve the wanted outcomes from stimulation.

### 1.3. Objectives

The objectives are to identify the following for the purpose of creating individualised models and personalised tDCS protocols that may be applied to effectively reduce or remove tinnitus

  (i). the stimulation parameters reported in current modelling studies of tDCS,
 (ii). the different approaches and steps taken in current modelling,
(iii). the predicted current intensity measured at the cortical level.

## 2. Materials and Methods

The protocol for this review follows the framework set by Arksey and O’Malley [17] and the Preferred Reporting Items for Systematic Reviews and Meta-Analyses Extension for Scoping Reviews (PRISMA-ScR) Checklist [18]. The review is registered with The Open Science Framework (OSF) and can be accessed online (https://doi.org/10.17605/OSF.IO/7G5R3).

### 2.1. Eligibility Criteria

The included records were required to involve the use of current modelling for tDCS based on real human data from participants aged 18 years and over (Table 1). The age restriction was decided to remove the added complexity of developmental changes in the brain and skull, and their impact on electrical activity. The grey literature was included (i.e., non-peer-reviewed articles such as theses, clinical trial protocols, and conference abstracts). Reviews were not included as the primary sources were likely to be retrieved from databases.

Stimulation required the involvement of conventional cortical tDCS. Studies of cerebellar tDCS, spinal tDCS, and deep brain stimulation were excluded as they do not target the cortical structures investigated in tinnitus and cannot be applied to the treatment of tinnitus. Studies of HD-tDCS were excluded because there is no evidence for its effectiveness over conventional tDCS [19]. It was also required that the included records be published in English. There was no exclusion for existing health conditions. There was no exclusion of records that did not investigate tinnitus, as the model construction pipeline for any condition could be applied to tinnitus by adjusting the targeted area and investigating outcome measures specific to tinnitus following stimulation.

### 2.2. Search Strategy

The search strategy was developed to procure as many relevant records as possible following the style and number of Boolean operators and wildcards offered by each database (Table 2). The following databases were searched: Medline (including AMED, APA PsychInfo, OVID, and Embase), PubMed, Web of Science, Cochrane Library, CINAHL ultimate, Scopus, IBECS, WPRIM, Science Direct, LILACS, KoreaMed, and CNKI. When searching Google Scholar to obtain part of the grey literature, all references would be gathered from every page with a record meeting the eligibility criteria until three consecutive pages without relevant records were passed. Google Scholar generated thousands of references and sorted them by relevance. This prevented the excessive inclusion of irrelevant records. The gathered references were then carried forward for title and abstract screening.

### 2.3. Title and Abstract Screening

Screening was conducted in Covidence by KT, BL, RSD, MS, and DJH. The reviewers followed the eligibility criteria (as summarised in Table 1), and each record was screened by two reviewers independently. Any conflicts were discussed and resolved between the two reviewers to reach an agreement. If there was no information to determine whether the record fit the eligibility criteria, the record was passed to full-text screening. This was initially piloted on three records to test for consistency and clarity of inclusion and exclusion criteria before formal screening began.

### 2.4. Full Text Screening

Two reviewers independently assessed the eligibility of the records based on the inclusion and exclusion criteria. KT, BL, RSD, MS and DJH were involved in full-text screening. The reasons for record exclusion were documented and are summarised in Figure 1. All texts deemed eligible by two reviewers were included for data extraction.

### 2.5. Data Charting

Data extraction was conducted by KT, BL, MSD and RSD. Two reviewers independently extracted items by following a data extraction form designed in excel specifically for this review. Before the review began, the data extraction form was tested in a selection of three papers for efficacy and sufficient detail. Following discussion among authors, redundant items were removed, and additional items were added or clarified before the formal data charting process took place.

### 2.6. Data Items

The following items were extracted based on the aims set out at the beginning of the review. These items were included to gain an understanding of the parameters set in current flow models, the software used, and the peak electric field magnitudes that were being recorded at the cortical level. This gave an indication of the peak electric field magnitudes that would result from different levels of applied current intensity. Items pertaining to study characteristics and design included author, year of publication, country of publication, type of study, aims, region of interest (ROI), and rationale for selected ROI. Population/sample characteristics extracted included the condition or context, sample size, age of participants, and sex of participants. Items on data acquisition were extracted, including data origin, image modality, resolution and weighting of scans. Parameters extracted were electrode montage, electrode dimensions and materials, factors affecting induced electric field (e.g., electrode orientation), current direction (anodal or cathodal), applied current intensity, and dose calculations. The modelling pipeline was investigated by extracting items including modelling software/toolboxes, tissues selected for segmentation, target current intensity, and mesh details. The results of the included studies were extracted based on the range in peaks of current intensity measured at the level of the cortex (V/m), peak current intensity and location at cortical level (V/m), range of current intensity necessary to achieve target intensity at ROI (V/m), changes or improvements in outcomes, and main conclusions.

## 3. Results

The data extraction table can be found in the Supplementary Materials and documents all information from the records pertaining to the data items. All software and software versions reported in the reviewed records are also available in the Supplementary Materials.

### 3.1. Selection of Sources of Evidence

Following screening and full text retrieval, 114 records were included for review. The process of study selection is summarised in Figure 1, along with the reasons for exclusion. 4782 records were gathered in total from the search. Four thousand seven hundred and eighty-two of these records came from online databases and include 4186 records from the initial search completed between May and July 2024, and 67 records from an updated search completed in August 2025. Five hundred ninety-six records were included from Google Scholar (501 from the initial search and 95 from the updated search). Two thousand three hundred and seventy-eight of these records were removed as duplicates. Two thousand four hundred four records were included for title and abstract screening, from which 2148 records were removed. The remaining 256 records were sought for retrieval, and of those, 2 were unavailable. Two hundred fifty-four full texts were screened for eligibility, and 140 were excluded, leaving 114 relevant texts for data extraction.

**Figure 2:**
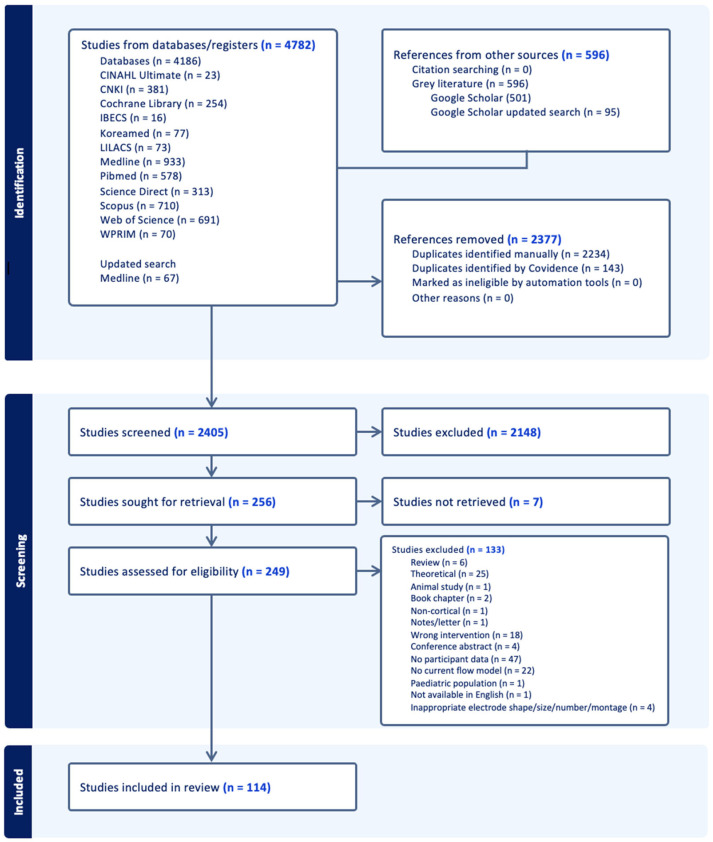
Flow diagram demonstrating selection of evidence [AMED = Allied and Complementary Medicine Database; APA = American Psychological Association; CINAHL = Cumulative Index to Nursing and Allied Health Literature; CNKI = China National Knowledge Infrastructure; IBECS = Indice Bibliográfico Español en Ciencias de la Salud; LILACS = Latin American and Caribbean Health Sciences Literature; WPRIM = Western Pacific Region Index Medicus].

The most common reason for records being removed during full-text screening was that they did not include participant data (i.e., current flow models were created but were not generated from human data), which resulted in the exclusion of 46 records. Twenty-five records were excluded as they were purely theoretical (i.e., there may be new developments in modelling techniques, but no models were generated in the study) and were not applied to participants. There were also 22 records that did not include details of a current flow model (Figure 2).

### 3.2. Characteristics of Sources of Evidence

Ninety of the included records were computational, and twenty-four were interventional studies, which include models based on the applied stimulation to predict the outcomes, or to explain the results post hoc. This included four case studies (three interventional [20,21,22] and one computational [23]), which investigated the use of tDCS in patients with very specific morphological differences due to pathology.

The country of publication was determined by the first affiliation of the first author. Countries producing the most records were the USA with 33 records (29%), and Germany with 14 records (12%). Finland and the Republic of Korea had eight records each, Iran and the UK had six, China, India, Japan and the Netherlands each had four records, Australia and Norway each had three records, Belgium, Brazil, Canada, Denmark, Italy, and Singapore each had two, and Czechia, France, Hong Kong, Russia, and Switzerland each had one. All included records were published between 2008 [24] and 2025.

Records were categorised based on the origin of the brain imaging data used in the models, and whether the models were individual or based on group data. They were classified as ‘primary’ if the scans were collected specifically for the study, ‘secondary’ if the scans were collected for a different study, or ‘database’ if the data were obtained from a database. The databases and datasets utilised by the reviewed records are presented in Table 3. Of the final included studies, there were 47 primary individual, 8 primary grouped, 15 secondary individual, 8 secondary grouped, 24 database individual, 7 database grouped, 4 mixed individual, and one not reported. It was more common that individual heads (86 records) were modelled than averaged heads (23 records). This may be because models based on the finite element method (FEM) are especially useful in reflecting individual differences. Averaged heads were created in the event that a demographic feature was being investigated, such as age [25] or ancestry [26], in which representative heads were created for people sharing a characteristic. Otherwise, there was no need to use grouped heads as the value of FEM models comes from their ability to accurately model individual morphology.

### 3.3. Region of Interest (ROI)

The region of interest is defined as the target area for neuromodulation, where we would expect to induce functional change, and is distinct from the electrode montage. The montage is the location of electrode placement, which is not always directly over the ROI. Stimulation and changes to the electric field could occur in the area outside the electrodes, or possibly between them. Although there was a large overlap, the electrodes may not necessarily be placed directly over the ROI.

The most common regions of interest investigated were the dorsolateral prefrontal cortex (DLPFC) and the primary motor cortex (M1). Thirteen studies specifically targeted only the DLPFC [20,28,29,30,31,32,33,34,35,36,37,38,39]. Thirteen records investigated only the primary motor cortex [40,41,42,43,44,45,46,47,48,49,50,51,52]. A further ten records investigated only the hand knob of the motor strip, specifically [13,14,53,54,55,56,57,58,59,60]. Many of the remaining studies investigated multiple brain areas, which may include the DLPFC and M1 [61]. The DLPFC and M1 areas may have been investigated more frequently because they are so well documented in previous studies of tDCS and can serve as a point of comparison for optimisation attempts. These studies were used to study variability in healthy participants, as well as the impact of pathology on morphology, such as cannabis use disorder [36] and the implications of these morphological differences on tDCS-induced E-fields. They may have used the DLPFC as an ROI because it is implemented in depression and provides an ideal target site for its treatment [38]. Studies which targeted the motor cortex may have involved conditions affecting the motor cortex or used movement-related outcome measures to assess the impact of stimulation. Fifteen records did not specify an ROI, and three records reported the whole brain, partly because the condition studied (e.g., chronic pain or migraine) is widespread [62] or to investigate the current distribution throughout the head [24,63].

### 3.4. Conditions and Contexts of Study

Many different conditions and contexts were studied using the current models. Pathological uses included Alzheimer’s disease, arthralgia, stroke, Parkinson’s, major depressive disorder, methamphetamine use disorder, mild cognitive impairment, glioma, dementia, ageing and age-related atrophy, schizophrenia, epilepsy, cannabis use disorder, obesity, bipolar disorder, and aphasia. The models were used to investigate the impacts of pathology on E-fields, to predict stimulation outcomes for a particular pathology, and for post hoc analysis to assess associations between stimulation effects and clinical outcomes.

None of the reviewed records applied tDCS to tinnitus and therefore cannot be directly applied to designing personalised tinnitus protocols. This also means that we cannot ascertain how the applied parameters impacted tinnitus-related outcomes. However, there are records that share the target of the DLPFC [20,28,29] and investigate dose control [13] as a means of considering inter-individual differences in anatomy. They also document modelling pipelines that could be applied to tinnitus. In future studies, these methods could be applied to create individualised models for participants with tinnitus, calculate individualised current doses, and investigate the impact of tinnitus-related outcomes compared to a fixed dose.

The four case studies included a case of left hemispheric stroke resulting in Broca’s aphasia, which demonstrated that lesions and perilesional areas have a significant influence on the induced E-field [64] and must be considered in protocol design. Two case studies use tDCS for successful rehabilitation after stroke [21,22]. Another case study explored the safety and outcomes of tDCS on a patient with frontotemporal lesions and schizophrenia, which revealed similar current distribution but lower peak intensities compared with a non-lesioned schizophrenia patient [20]. These case studies all reflect the value of modelling based on individual data, especially for individuals with more complex and pathological cases, as these differences can impact tDCS-induced E-fields.

Alzheimer’s disease was associated with reduced field intensities in the ROI [31], which may suggest a higher current dose is needed to achieve the same effects on E-fields as in individuals without Alzheimer’s. This reduced current intensity may be related to increased relative volume of CSF and brain atrophy [65], causing current to be shunted or redirected away from target areas.

Multiple studies highlighted the importance of using individualised models to predict tDCS effects on E-field to account for individual differences in morphology and to personalise stimulation, particularly when designing protocols for more complex cases involving metallic instrumentation [22] or tumours [42] to allow for wider access to tDCS. In cases investigating tumours, it was found that tDCS application was safe, but tumour presence alters the current distribution during tDCS [66], emphasising the importance of modelling to account for differences in morphology on E-fields. Additionally, current flow models were used to investigate the option of using tDCS to modify tumours before surgery as a protective measure against potential loss of function due to surgical damage [42]. This use can be combined with motor training to preserve the global connectivity of the sensorimotor network [45].

Studies of white matter lesions were performed to assess their impact on the E-field, and established a general pattern of increased E-field magnitude in the ROI for lesions oriented along the predominant direction and decreased E-field magnitude for lesions lying in the opposite direction [67]. This was consistent across ROIs and individuals, with a greater impact if the lesion was closer to the ROI and larger in volume. Smaller lesions further from the ROI had a minimal impact on E-field magnitude and can be ignored from models if the lesion load can be classified as low to medium [68]. When modelling white matter hyperintensities, it was found that a higher applied current intensity was necessary to achieve the equivalent measured current intensity in non-lesioned tissue [69].

In the context of healthy participants, modelling was used to understand cognitive processes such as working memory [70] and language production [71]. Healthy participants were used to model example individuals for tDCS optimisation, i.e., to investigate differences in outcomes based on changes to electrode montage. Modelling of healthy participants can also be used to develop new modelling techniques and simulate pathology to create tools that can be applied to patients with those conditions. Laakso developed a new method for analysing electric fields at the group level [72] in a standard brain space for post hoc analysis of existing modelling studies, which allows for other researchers to gain access to a wide range of stimulation modelling data for their own analysis. Machine learning has been used to predict whether an individual is likely to be a responder or non-responder based on applied current intensity, with 86% accuracy [70]. Here, Albizu et al. [73] suggested that non-responders would require an average increase in applied current intensity of 4.3 mA (range 3.19–5.37 mA) to match the profile of responders, but in models, applied a dose of 2 mA. This recommendation of applied intensity exceeds any of the current doses applied in the reviewed studies and is at a level that may require consideration in terms of safety limits. In a review by Bikson et al. [74] it was concluded that in human trials using conventional tDCS, no Serious Adverse Effects were reported for stimulation ≤40 min, ≤4 mA, ≤7.2 Coulombs in 1000 participants, even with repeated sessions and in vulnerable populations.

### 3.5. Factors Influencing tDCS-Induced Electric Field

The records included in this review confirm the presence of inter-individual variability and emphasise the value of generating individualised models for accurate prediction of E-fields and reduced outcome variability [28,43,73].

The impact of sex on electric fields was a factor that was explored in the included studies but was not an isolated factor for investigation. Sex was explored in the context of age-related individual differences. It seems that the impact of sex differences depends on the age of the individual. Bhattacharjee et al. [25] investigated the impact of sex differences in three age groups: young (18–40 years), middle (41–63 years), and older (64–87 years). They observed that in the young group, males received greater current intensity than their female counterparts. In the middle group, there were no differences in current density. In the older group, females received greater current density than their male counterparts. Similarly, Kashyap et al. [75] observed that the relationship between E-field and applied current can be linear or non-linear and was influenced by age; non-linearity was more prominent in older adults and more likely as age increased. They reported that generally, there was a decrease in field focality with age which was more evident in males over forty years of age and suggested using a higher current dose for these groups to achieve the same results that would be expected from 2 mA applied to a younger adult (under forty years of age), which reiterates the presence of sex differences in older adults and the need to consider age and sex when personalising protocols.

A study by Indahlastari [76] used a sample aged 51–95 years (mean 73.9 years) and inversely correlated the predicted current intensity with brain atrophy, which is associated with increased age and relative CSF volume. This result is consistent with previous studies explaining the influence of high relative CSF volume on current shunting [14], which resulted in current flow reaching the target area with a lower intensity than can be expected in a young adult. Here, the authors recommended increasing the current dose to exceed 2 mA to account for age-related brain atrophy. Additionally, current intensity was reduced in older adults with cognitive impairments, posited to be the result of age-related atrophy and increased relative CSF, which has implications when applying tDCS to individuals with Alzheimer’s [46,65]. Again, the applied current intensity may need to be increased in this context to produce the desired stimulation outcomes.

Although ancestral background might have influenced morphology, when head models were averaged within three ancestry groups (European, Chinese, and Indian), the variability in current flow patterns did not exceed the variability between groups compared with the variability between individuals [26]. This suggests that ancestry is not a sufficiently robust characteristic to predict stimulation outcomes or to inform the design of individualised protocols.

### 3.6. Electrode Montage, Materials, and Dimensions

Conductivity values were typically kept to the default settings provided with the software, which were based on neurophysiological studies [77]. Forty-one records modelled 5×7 cm or 35 cm^2^ electrodes. Thirty-two records modelled 5×5 cm or 25 cm^2^ electrodes. Disc electrodes in the with a range of radius from 0.25 cm [52] to 3.38 cm [78] were modelled. Point electrodes [79] and triangular electrodes (10 cm base, 7 cm height) [78] were also modelled. When using triangular electrodes, it was recommended to use an empty triangular montage for increased electric field intensity and current density.

In terms of placement, there were no significant changes to the electric field when the electrodes were moved by 1cm from the original position [80] across three different electrode montages, F3–F4-1, F3–F8 and F3–EC. However, changes large enough to result in different montages were followed by the stimulation of different structures and different depths of current distribution [81]. Larger changes in electrode placement from the original position may also influence the depth of stimulation. For instance, fronto-extracephalic or fronto-occipital montages resulted in current flow to deeper structures such as the anterior cingulate cortex compared to F3–F4 and F3–F8 montages [80].

The distance and size between electrodes influenced the E-fields. Smaller electrodes allowed for higher focality, and they also led to increased variability in the electric field distribution [55] that should be considered when designing protocols. In addition, small electrodes generated higher current intensity and current density; the focality was difficult to control. Alternatively, the larger electrodes were associated with decreased current density, but focus was easier to manipulate.

Although one electrode is often referred to as ‘active’ and the other as a ‘return’, both were reported to have an impact on the E-field distribution [82]. Therefore, it is important to consider the placement and dimensions that each will have when designing montages. Additionally, it was suggested that electrodes should be accurately modelled where possible and be consistent with the electrodes used in actual stimulation in terms of location, distance, dimensions, and contact area for the generation of accurate models and resulting E-field predictions [83].

### 3.7. MRI Acquisition

Generally, MRI acquisition was very consistent. All included studies used an MRI to generate the individualised head model, using at least a T_1_-weighted anatomical image was used. Many studies also combined T_2_-weighted scans with the T_1_-weighted anatomicals, which was purported to improve the robustness of segmentation, particularly for accurate skull segmentation [84], and recommended when using ROAST and SimNIBS. In most cases, these scans would be converted from DICOM to NIfTI format with a tool such as dcm2niix [85] prior to segmentation.

### 3.8. Segmentation

In more recent studies and since the introduction of automated image segmentation, many of the included studies segmented the same tissue types from the input MR images. These included white matter, grey matter, cerebrospinal fluid, bone/skull, scalp/skin, and air (such as cranial foramina and the orbits). In 20 studies, it was reported that the bone was subdivided into spongy and compact bone, or cancellous and cortical bone, which better represents the consistency of the head and could provide improved accuracy in electric field modelling. Studies using the default conductivity values from SimNIBS and ROAST would have also accounted for fat and muscle, whereas other studies may model soft tissue (i.e., muscle, fat, skin) simply as one tissue type.

Records generally implement automated segmentation. This process may have involved using SPM [86] (used in ROAST), Headreco [87] (for versions of SimNIBS before 4.0.0.), or Charm [88] (for versions of SimNIBS from 4.0.0 onwards). A common step was to visually inspect the segmentations in FreeSurfer [89] or the SimNIBS charm report for consistencies between the tissue boundaries with a template or the original MRI overlaid. The segmentations were manually corrected if necessary.

### 3.9. Modelling Pipeline

There was less deviation from the standard pipeline than we would expect given that the available modelling software allows extensive flexibility in defining input parameters. The standard pipeline involved converting the MR images into a suitable format for modelling (if not already in NIfTI format), i.e., from DICOM to NIfTI. This conversion step is well documented and a common step [85] when working with MRI. This is generally followed by automatic segmentation, mesh generation, and solving of the finite element model. The tissues are segmented, and their conductivities are usually unchanged from the default options of the software package used. Only five of the reviewed records mention anisotropy [24,50,61,90,91], which implies that the direction of current and the orientation of the E-field were not considered to the same extent as factors such as electrode montage and current dose. It was reported that white matter anisotropy could affect the predicted current intensity at the white matter and grey matter by 36% and 8%, respectively [91], which suggests that the inclusion of anisotropy in models has a greater impact if the target region is a deeper structure rather than a structure of the cortical surface information and may be a useful component of current models.

The use of SimNIBS was reported in 42 (37%) records. ROAST was used in 23 (20%) records. There were three studies that used both ROAST and SimNIBS packages [39,92,93]. In software such as ROAST, there are options to modify the generated mesh. ROAST calls on iso2mesh for finite element model mesh generation, making it an automatic step in this pipeline. SimNIBS makes use of Gmsh [94] to create a finite element mesh and visualise the 3D model. Both toolboxes are used for the same purpose but are built into the software packages, meaning that user preference in toolboxes may determine which pipeline is utilised. The mesh parameters can be specified, but specific mesh settings were only reported in one of the reviewed records [13]. These parameters can be used to manipulate the maximal element size, minimal angle of a surface triangle, maximal radius-edge ratio, and maximal tetrahedral element volume. It seemed that for standard use, the default mesh settings are sufficient. It was reported that while these two pipelines produce slightly different electric fields, one is not superior to the other, but more evidence is needed to validate whether one produces more accurate predicted E-fields than the other [92]. The appropriateness of a particular pipeline may also depend on the intended use.

Other pipelines were used, which followed similar steps to create individualised models. An example of this [82] used Simpleware™ (Synopsys, Inc., Sunnyvale, CA, USA) ScanIP for segmentation, Simpleware™ ScanFE to convert segmented tissues to volume meshes and set boundary conditions, and finally solved the finite element model in COMSOL MULTIPHYSICS^®^ (COMSOL, Inc., Burlington, MA, USA) [95]. This followed the same steps as automated pipelines and was conducted before the introduction of ROAST or SimNIBS. Twenty-six studies followed the same or similar sequence using Simpleware and/or COMSOL.

### 3.10. Applied Current Intensity

A persistent pattern throughout the included studies was that increased applied current intensity was accompanied by increased predicted current intensity in the ROI and functional connectivity [42,96]. However, the current intensity was applied within a relatively small range. In one study [73] applied current intensity ranged from 0.1 to 4.0 mA. However, no peaks in measured current intensity were reported. Outside of this study, the applied current intensity ranged from 0.5 [20,44,57,97]–3.0 mA [97,98,99]. Ninety-two studies (81%) applied either 1 or 2 mA. Forty-five records (39%) reported applying 2 mA only, and 47 (41%) reported applying 1 mA only. The applied current intensity can be found with its respective peak electric field magnitude in the Supplementary Materials, as well as the main findings and changes in outcomes.

Studies applying higher current doses were non-interventional [73] (0.1–4.0 mA), [75] (1–3 mA), which does not allow for the observation of adverse effects and therefore cannot give insights into the safety limits of higher current doses. Two interventional studies exceeded the application of 2 mA [73,100], neither of which reported any adverse events. Both applied a maximum of 3 mA and reported improvements in task performance compared to sham conditions.

### 3.11. Target Current Intensity

To inform the current dose, having a target current intensity at the level of the cortex is valuable in determining whether the current applied and measured is sufficient or optimal to achieve the desired outcomes of stimulation. However, there was no consensus on a target current intensity necessary to drive neuroplastic change and reach the desired stimulation outcomes. In studies that defined a target current intensity, this was the mean of the group [13] or an arbitrary number used as a target for reducing variability [101].

Four studies [13,68,75,102,103] included the following dose calculation equation (or equivalent), which will be important in creating personalised protocols to reduce variability in stimulation outcomes:

$$Individualised\;current\;dose=\frac{Target\;electric\;field\;magnitude}{Actual\;electric\;field\;magnitude}\;\times \;Fixed\;dose$$

Individualised current dose and fixed dose are in mA. Actual electric field magnitude is the achieved current intensity when using the fixed dose and is in V/m. The target electric field magnitude is the desired current intensity at the cortical level and is in V/m.

Evans et al. [13] used the group mean was used as a target current intensity with the aim of reducing variability in measured peak current intensity. The variability was reduced when using an individualised dose and produced a range in current intensity at the ROI of 0.176–0.190 V/m compared to a range of 0.353–0.386 V/m using a fixed dose. This study highlighted the impact of dose control on variability and offers a solution to make tDCS a feasible treatment option for tinnitus.

### 3.12. Peak Current Intensity

Sixty studies (53%) did not report the range in peak current intensity measured at the level of the cortex or did not report in V/m. Current intensity and distribution may be reported by field percentiles or field focality, which are automatically generated when using SimNIBS. Where possible, values were converted to V/m for comparison. However, reported values of the current distribution could not be converted directly. Mean values in peak current intensity were disregarded during data extraction to focus on individualisation of protocols.

In studies that assessed the stimulation outcomes with the involvement of models to simulate the electric fields during actual stimulation, it was reported that current intensity in the ROI was the strongest predictor of treatment response. Albizu et al. [70] applied F3/F4 stimulation to healthy adults with 2 mA intensity and observed improvement in the N-back test post-intervention compared with pre-intervention in responders to tDCS, which corresponded with improvements in working memory performance. They also used individual, MRI-derived current models to develop a machine learning algorithm that could predict whether a participant would respond with 86% accuracy, considering two crucial characteristics of the individual electric field—intensity and direction. The authors highlighted that individual differences were a key influence on current intensity and direction of stimulation outcomes, which lends support for the use of individualised models in personalised tDCS protocol design.

The peak current intensity measured at the cortical level ranged from 0.090 V/m [58] to 2.44 V/m [32]. There were only five studies that applied current intensity exceeding 2 mA, and none of these reported the induced electric field peak magnitude. As a result, we cannot deduce the impact of stimulation intensities higher than 2 mA on the electric field magnitude or whether intensities exceeding 2 mA result in improved efficacy.

### 3.13. Analysis and Visualisation

ROI analysis was used in the records [104,105] to average the absolute peak values within an ROI by applying tissue masks to isolate a sphere around the desired MNI coordinate or using surfaces and an atlas to specify a region. This reduced the impact of abnormally high absolute peak values in electric field magnitude and allowed for a more realistic indicator of electric field strength to be obtained. This requires analysis outside of the GUI in Matlab or Python on the output files, but is supported by pre-written scripts available in documentation from ROAST and SimNIBS authors.

## 4. Discussion

The reviewed studies give many insights into current models of tDCS, highlighting factors that influence the electric field but also the accuracy of models. Applied current intensity was associated with greater reported current intensity within the ROI and better performance in post-intervention tasks. It can be inferred that age and its interaction with sex have a significant impact on tDCS-induced electric fields, and this can be attributed to age-related differences in anatomy. The included studies have also highlighted the importance of individualised modelling reflecting actual stimulation as closely as possible to provide accurate and realistic predictions of E-fields that are valuable in designing personalised stimulation protocols. It has also been established that pathology affects current distribution, which has not yet been investigated using models for individuals with tinnitus and is a potential area for further investigation.

### 4.1. Data Origin

Individual models were far more prevalent in the reviewed records than were grouped models. This may be because averaging across multiple heads minimises the impact of considering anatomical differences using MRI data. It may be useful to average MRI data in training machine learning algorithms to guide protocols. Another context in which heads were averaged was when comparing samples representing specific populations with shared characteristics, such as age [63,106] and ethnicity [26]. This allows for the consideration of common characteristics between populations rather than between individuals and could also be implemented to investigate differences in outcomes between sexes, conditions, and contexts.

### 4.2. Individualised Head Models

Modelling individual heads is repeatedly stressed by authors of the reviewed records for accurate and personalised predictions of electric field intensity and distribution. They emphasised how valuable realistic heads are for each individual in reflecting their morphology and how current would flow for them based on their individual anatomy, highlighting the presence of anatomical variation and the impact that different tissue distributions have on the resulting current distribution. They also highlight the importance of accurate and realistic modelling to reflect the different conductive properties of the different tissues, with more recent studies including. However, fat, muscle, and skin all vary in morphology and conductivity values [107,108], so combining them oversimplifies the resulting model. For more realistic predictions, it is therefore important to accurately model tissue types to account for the impact of different conductive properties on current flow.

### 4.3. Stimulation Parameters

There was a relatively small range in which current intensity was applied in current flow models from the included records. Most studies used only 1 or 2 mA. It was emphasised that for older individuals or for those with complex brain morphology, it is especially important to create individualised models for accurate representation of E-fields to guide personalised tDCS protocols. Although it was encouraged to use higher doses in older individuals, there was little information on the upper limit for the current dose or the safety limitations, which could be further investigated.

In studies that investigated dose variation as a means of optimisation [13] it was reported that there was a linear relationship between applied current and predicted current intensity, but also that this linearity was more likely to occur in individuals under 40 years of age, and that with increased age, there was an increased likelihood of this relationship being non-linear [75]. However, none of the studies investigated this in participants with tinnitus, which is an area for further exploration.

In tinnitus, findings from studies on electrode placement may be useful in targeting deeper structures of the limbic system, which maintain the emotional response to aberrant sound perception. Further investigation of montages could be performed to assess the efficacy of deeper stimulation montages to modulate the emotional aspect of tinnitus.

### 4.4. Impacts for Non-Programming Experts

The development of automated modelling software makes current flow models more accessible and allows experts with an understanding of neurophysiology, neuroanatomy, and pathology to plan their tDCS protocols without extensive training in programming. This software can also model using default conductivity values based on physiological studies [77] and may include a graphical user interface (GUI) which makes electrode placement simpler than creating custom scripts and permits visualisation before running the simulation, allowing the user to check the placement in the model against actual placement on an individual. SimNIBS and ROAST will even package multiple toolboxes such as SPM, Headreco, and Charm for segmentation, iso2mesh [109] (for ROAST) and Gmsh (for SimNIBS) for mesh generation, and getDP [110] as a finite element solver, allowing for multiple steps to be completed in one place.

Many steps in generating current models have been made automated, which has significantly improved accessibility for those who do not have programming knowledge. There is still a learning curve in using these software packages and becoming familiar with the general process involved in modelling; there is no longer a need to learn how to create a mesh, to manually segment MR scans, or to perform field calculations. The presence of a graphical user interface further adds to the accessibility of modelling and reduces the coding needed to create models while allowing models to be customizable when setting parameters. The only need for knowledge in more technical aspects of modelling when using these packages is to troubleshoot when models are not created as expected. Nonetheless, there is support even from open-source software packages in resolving modelling issues. There is extensive documentation to support and explain the process of current flow modelling using these software packages.

Recommendations for non-programmers

1. Use an open-source package such as SimNIBS or ROAST. These packages bring together and automate all the toolboxes necessary to complete the modelling pipeline and provide a wealth of support, datasets, tutorials, and documentation with few inequities in software accessibility. If there is a preference or possibility to use proprietary software, it is advisable to use a package that encompasses and automates as much of the process as possible, such as Neurophet tES Lab, and provides extensive support for users.
2. Make use of the package documentation. The authors provide comprehensive instructions that can be followed without programming knowledge, as well as pre-written scripts for the standard uses of the packages.
3. For segmentation, use both T_1_- and T_2_- weighted anatomical MR images and convert them to NiFTI format (can be performed using software such as MRIcron v1.0 or MRIcroGL v1.2). This produces a more robust segmentation than using only T_1_-weighted images, particularly for the boundaries of the skull.
4. If the software allows, check the segmentation visually for errors and correct if necessary (e.g in FreeSurfer).
5. Make use of the GUI. Use of a model setup which is consistent with active stimulation. The GUI allows the user to visualise electrodes to ensure they are modelled with the same orientation, position and size that may be used on participants.
6. Use the default conductivity values, as these are based on physiological studies of tissue conductivity. Similarly, use the default mesh settings as these are sufficient for standard use.
7. Set the electrode dimensions, position, and orientation as they would appear for active stimulation. This includes defining whether gel or saline is used, and the thickness at which they will be applied.
8. Minimum reporting includes the peak electric field magnitude and current density.

### 4.5. Outcome Reporting

Several data items were largely unreported. These included details of dose calculation, target stimulation intensity at the cortex, and the necessary range in current intensity necessary to achieve the target stimulation at the cortical level.

It is possible that the target current intensity may exist within a range or that the current intensity needed to achieve the desired outcomes is variable between individuals. Dose response has been effective in reducing variability with available calculations. As previously discussed, it has been used to achieve a range in current intensity at the ROI of 0.176–0.190 V/m compared to a range of 0.353–0.386 V/m using a fixed dose [13]. Future work to personalise tDCS for tinnitus could be advanced by implementing these calculations with models, and may give some indications of a target current intensity sufficient to effectively reduce tinnitus symptoms.

There were differences in the methods in which the results were reported. Peak electric field magnitude was not often reported as an absolute peak in V/m for each individual head model. It was also reported as a group peak average, a mean within an ROI or whole brain. There were a few records that reported details pertaining to meshing, largely because the software they were using had automated this process. This change from manual meshing is an improvement for modelling efficiency.

When reporting a peak, it may be unrepresentative of the physiological effects on a larger scale, especially if the peak only pertains to a single voxel. In this context, it would be useful to also calculate the peak within a region of interest, which some studies included in addition to reporting absolute peaks [104,105].

### 4.6. Inter-Individual Differences

The included records confirmed the presence of variability between individual E-fields. Age and its impact on morphology seemed to be an important influence on tDCS-induced E-fields and should be carefully considered when designing protocols. Increased age was associated with higher variability in electric field magnitude, likely due to increased relative volume of CSF and the resulting redirection of current flow. It was also highlighted that this variability with increased age was more prevalent in men. Consequently, numerous authors suggested applying current intensity exceeding 2 mA in older participants (the authors suggest the cutoff to be over 40 years) [75] to achieve the same results as one might expect for a young adult. However, the authors do not suggest upper limits for stimulation, which may be a concern for the likelihood of adverse events

### 4.7. Future Directions

Several factors could be further explored in creating more effective and personalised current flow models. There was a lack of evidence pointing to a target current intensity at the level of the cortex necessary to achieve optimal stimulation or for the desired outcomes. The absence of a target in the electric field intensity makes it difficult to set a dose for applied current intensity. A target current intensity could be established for tinnitus by correlating outcome measures such as tinnitus loudness with predicted current intensity following actual stimulation and modelling the conditions of the stimulation. This may allow for reverse calculation and dose control to achieve more consistent current intensity.

When investigating optimisation and personalisation, dose control was not largely the study focus, which is an area for further investigation in personalising tDCS to reduce variability in outcomes. None of the reviewed studies investigated the impact of dose control for individuals with tinnitus, which could be a subject for future work.

Only eight studies included cathodal tDCS [53,79,80,82,90,97,111,112] (i.e., current which runs from cathode to anode, leading to an overall decrease in cortical excitability), which gives little indication of the likely effects for current intensity applied outside of the 1–2 mA range, or the impact of dose-control for this polarity. In the context of tinnitus, cathodal stimulation is used to decrease levels of activity in regions of the brain that may produce phantom auditory perceptions or maintain the emotional response to them. Consequently, the results of studies using anodal tDCS must be carefully considered when applying them to the design of protocols using cathodal stimulation. Additionally, more investigation could be performed to assess the impact of dose control using cathodal stimulation.

### 4.8. Limitations

We investigated the efficacy of stimulation by the peak current intensity measured at the cortex. However, a high current intensity at the cortex may not be the only influence on the stimulation outcomes and does not predict how long the effects will last beyond stimulation. The current flow models only reflect the electric field induced during stimulation and not the offline effects, meaning they are not the sole determinant of stimulation effectiveness. The models cannot account for factors that may influence the likelihood of plastic changes and resulting long-term effects, such as contextual influences [113]. Hormonal influences may also affect plasticity and contribute to different levels of plasticity depending on the cycle phase and which hormone is being released [114]. Oestrogen has been associated with synaptogenesis between hippocampal neurons [115] and oestradiol release has been observed to induce the formation of new dendritic spines in hippocampal neurons [116]. Physical activity levels are another contributor to neuroplasticity, which cannot be included in the computational models, but influences the release of neurotrophic factors related to neuroplastic changes [117] and is worth considering in the design of personalised protocols.

Using peak values may be misleading if they occur outside of the target region or within a very small volume of the brain and do not produce significant differences in electric field magnitude or distribution. Some of the studies in this review remedied this by calculating the mean electric field magnitude within the ROI [104,105]. It may be beneficial to consider how other characteristics of the electric field impact the stimulation outcomes and how to control for endogenous influences on plasticity and the response to stimulation.

The exclusion criteria also introduced some limitations to the review. Only records written in English were included, as it was not feasible for the authors to translate other languages. This exclusion may have resulted in relevant records being omitted from the review, leading to an unrepresentative account of how current flow is modelled across the world. It is also possible that the data gathered is not representative of all efforts in current modelling for tDCS, as it may be impacted by publication bias. In limiting the review to records involving only adults, the complexity of early development of the skull and brain is removed as a factor in tDCS modelling design. However, this topic may be substantial enough to warrant its own investigation in the future.

## 5. Conclusions

This review has compiled the parameters and software used in numerous studies of current modelling for tDCS, establishing common practices in methodology and highlighting areas for further exploration in the development of personalised tDCS protocols for the treatment of tinnitus. It also reiterates the importance of models being consistent with actual stimulation parameters for accurate predictions or post hoc analysis of E-fields. Ultimately, the reviewed articles highlighted the value of creating individual current flow models for personalised tDCS to reduce inter-individual variability arising from age and sex-related differences in morphology and their influence on E-fields. These inter-individual differences may be present between individuals with tinnitus and should be considered in the modelling and protocol design for tDCS targeting areas implicated in tinnitus to achieve the desired stimulation outcomes, particularly when calculating the current dose.

## Figures and Tables

**Table 1:** Final inclusion and exclusion criteria.

Inclusion Criteria	Exclusion Criteria
Participants aged 18 and over	Participants under 18 years
Cortical tDCS	Non-cortical tDCS, other neuromodulation techniques, and HD-tDCS
Any year of publication	Review papers
Human participants	Animal models or purely theoretical/computational models
No exclusion based on existing health condition	
Written in English	Not published in English

**Table 2:** Search term strategies for tDCS current modelling methods [AMED = Allied and Complementary Medicine Database; APA = American Psychological Association; CINAHL = Cumulative Index to Nursing and Allied Health Literature; CNKI = China National Knowledge Infrastructure; IBECS = Indice Bibliográfico Español en Ciencias de la Salud; LILACS = Latin American and Caribbean Health Sciences Literature; WPRIM = Western Pacific Region Index Medicus].

Search Terms	Search Engine
(tDCS OR transcranial direct current stimulation) AND (computation* OR mathematical model* OR current flow model* OR current simulation OR ROAST OR SimNIBS OR FEM OR finite element*)	Medline (AMED, APA PsycInfo, OVID, Embase), PubMed, Web of Science, Cochrane Library, CINAHL ultimate, Scopus, IBECS, WPRIM
(tDCS OR transcranial direct current stimulation) AND (computation OR mathematical model OR current flow model OR current simulation OR ROAST OR SimNIBS OR finite element)	Science Direct
(tDCS OR transcranial direct current stimulation) AND (computational modelling OR mathematical modelling OR current flow modelling OR current simulation OR ROAST OR SimNIBS OR FEM OR finite element method)	LILACS, KoreaMed, CNKI, Google Scholar

**Table 3:** Databases included in the reviewed records.

Database	Reference
Alzheimer’s disease neuroimaging initiative (ADNI)	https://adni.loni.usc.edu/
Cambridge Centre for Ageing and Neuroscience (Cam-CAN)	https://opendata.mrc-cbu.cam.ac.uk/projects/camcan/
Human Connectome Project (HCP)	https://www.humanconnectome.org/. HCP, WU-Minn Consortium (Principal Investigators: David Van Essen and Kamil Ugurbil; 1U54MH091657) funded by the 16 NIH Institutes and Centres that support the NIH Blueprint for Neuroscience Research; and by the McDonnell Centre for Systems Neuroscience at Washington University.
Ischemic Stroke Lesion Segmentation (ISLES)	https://www.isles-challenge.org/
National Alliance for Medical Image Computing (NAMIC: brain multimodality)	https://www.na-mic.org/wiki/Downloads
Neurodevelopmental MRI database	https://www.nitrc.org/projects/neurodevdata/
Open fMRI database	https://openfmri.org/
Simulated brain database of Brainweb	http://www.bic.mni.mcgill.ca/brainweb/
Sleepy Brain Project	https://openneuro.org/datasets/ds000201/versions/1.0.3
XNAT database or Open Access Series of Imaging Studies (OASIS)	https://sites.wustl.edu/oasisbrains/ Open Access Series of Imaging Studies (OASIS): Cross-Sectional MRI Data in Young, Middle Aged, Nondemented, and Demented Older Adults [27].

## Data Availability

The original contributions presented in this study are included in the article/Supplementary Material. Further inquiries can be directed to the corresponding author.

## Supplementary Materials

The following supporting information can be downloaded at: https://www.mdpi.com/article/10.3390/brainsci16010044/s1. Data extraction; Preferred Reporting Items for Systematic reviews and Meta-Analyses extension for Scoping Reviews (PRISMA-ScR) Checklist.

## Abbreviations

The following abbreviations are used in this manuscript:

AMED	Allied and Complementary Medicine Database
APA	American Psychological Association
CENTRAL	Cochrane Central Register of Controlled Trials
CINAHL	Cumulative Index to Nursing and Allied Health Literature
CNKI	China National Knowledge Infrastructure
CSF	Cerebrospinal fluid
DICOM	Digital imaging and communications in medicine
DLPFC	Dorsolateral prefrontal cortex
FEM	Finite element model
GM	Grey matter
GUI	Graphical use interface
HD-tDCS	High definition transcranial direct current stimulation
IBECS	Indice Bibliográfico Español en Ciencias de la Salud
LILACS	Latin American and Caribbean Health Sciences Literature
M1	Primary motor cortex
MRI	Magnetic resonance image
NIfTI	Neuroimaging Informatics Technology initiative
ROI	Region of interest
SPM	Statistical parametric mapping
tDCS	Transcranial direct current stimulation
tES	Transcranial electric stimulation
WM	White matter
WPRIM	Western Pacific Region Index Medicus
